# *Mycobacterium tuberculosis* Genotypes Determined by Spoligotyping to Be Circulating in Colombia between 1999 and 2012 and Their Possible Associations with Transmission and Susceptibility to First-Line Drugs

**DOI:** 10.1371/journal.pone.0124308

**Published:** 2015-06-11

**Authors:** Gloria Puerto, Lina Erazo, Maira Wintaco, Claudia Castro, Wellman Ribón, Martha Inírida Guerrero

**Affiliations:** Dirección de Investigación en Salud Pública, Grupo de Micobacterias, Instituto Nacional de Salud, Bogotá, Colombia; Institut National de la Recherche Agronomique, FRANCE

## Abstract

**Introduction:**

Tuberculosis (TB) remains a primary public health problem worldwide. The number of multidrug-resistant tuberculosis (MDR TB) cases has increased in recent years in Colombia. Knowledge of *M*. *tuberculosis* genotypes defined by spoligotyping can help determine the circulation of genotypes that must be controlled to prevent the spread of TB.

**Objective:**

To describe the genotypes of *M*. *tuberculosis* using spoligotyping in resistant and drug-sensitive isolates and their possible associations with susceptibility to first-line drugs.

**Methods:**

An analytical observational study was conducted that included 741 isolates of *M*. *tuberculosis* from patients. The isolates originated from 31 departments and were obtained by systematic surveillance between 1999 and 2012.

**Results:**

In total 61.94% of the isolates were resistant to 1 or more drugs, and 147 isolates were MDR. In total, 170 genotypes were found in the population structure of Colombian *M*. *tuberculosis* isolates. The isolates were mainly represented by four families: LAM (39.9%), Haarlem (19%), Orphan (17%) and T (9%). The SIT42 (LAM 9) was the most common genotype and contained 24.7% of the isolates, followed by the genotypes SIT62 (Haarlem1), SIT53 (T1), and SIT50 (H3). A high clustering of isolates was evident with 79.8% of the isolates classified into 32 groups. The Beijing family was associated with resistant isolates, whereas the Haarlem and T families were associated with sensitive isolates. The Haarlem family was also associated with grouped isolates (p = 0.031).

**Conclusions:**

A high proportion (approximately 80%) of isolates was found in clusters; these clusters were not associated with resistance to first-line drugs. The Beijing family was associated with drug resistance, whereas the T and Haarlem families were associated with susceptibility in the Colombian isolates studied.

## Introduction

Tuberculosis (TB) remains a primary public health problem and is the second leading cause of death from infectious diseases in the world. According to the World Health Organization (WHO), 8.6 million individuals worldwide were infected with TB in 2012, and although TB is a curable disease, 1.3 million individuals died from TB infections [1].

A steady trend in the incidence of tuberculosis has been observed in Colombia since 1999, with an average of 25 cases per 100,000 inhabitants and 11,000 new cases reported each year [2]. Regarding the cases resistant to first-line anti-TB drugs, the latest national surveillance study of resistance conducted in 2004 and 2005 showed a prevalence of 2.38% (95% CI: 1.58–3.57) of multidrug-resistant tuberculosis (MDR TB) in untreated patients. Although this increase was not statistically significant relative to previous studies, it may have epidemiological value and constitutes a serious threat to TB control [3].

Spoligotyping is a molecular technique based on the characterization of the polymorphisms of the direct repeat (DR) locus found exclusively in members of the *M*. *tuberculosis* complex, and it is a simple, rapid and inexpensive typification method with results that can be compared among laboratories worldwide. Additionally, the SpolDB4 database (http://pasteur-guadeloupe.fr:8081/SITVITDemo) includes classifications for spoligotypes and descriptions of the genetic families of *M*. *tuberculosis* for 62,582 isolates from 153 countries; these isolates contain 7105 patterns of spoligotyping that are grouped into 2740 SIT (Shared International Type) codes [4]. The characterization of isolates can be used to identify patients with identical genotypes, which may be potentially associated with the same transmission path, in contrast to ungrouped genotypes that originate from reactivation or latent infection [5]. Molecular epidemiology information is useful in the context of epidemic events and the transmission of tuberculosis. Some studies have reported the establishment of optimal treatment schemes for patients with identical isolates identified by spoligotyping compared with other grouped strains that were previously associated with MDR TB [6].

The evolution of the DR locus has enabled the analysis of population structures, and this approach can be used to classify *M*. *tuberculosis* complex in lineages or families [7–10]. The DR-based approach clearly reveals two major lineages (1 and 2) that contain various families or sublineages; for lineage 1, the families are: African (Uganda, Cameroon and S), Asian (Beijing and CAS), Latin American-Mediterranean and African-European (X, Ghana and Haarlem); for lineage 2, only the EAI family affects humans, whereas the *M*. *bovis*, *M*. *caprae* and *M*. *microti* families primarily affect animals [11].

This study aimed to describe the genetic diversity of *M*. *tuberculosis* by spoligotyping 741 clinical isolates obtained from 1999 to 2012 and to determine their possible associations with transmission and susceptibility to first line drugs in Colombia.

## Materials and Methods

### Type of study

An analytical observational study was conducted to evaluate the possible association of *M*. *tuberculosis* genotypes identified by spoligotyping a group of Colombian isolates with susceptibility to first-line drugs (rifampicin, isoniazid, streptomycin and ethambutol); similarly, demographic, clinical and epidemiological variables were assessed. This study included isolates belonging to the *M*. *tuberculosis* complex that were obtained from 31 departments in Colombia between 1999 and 2012; these isolates were collected through systematic surveillance performed by the National Institute of Health, the INS.

### Sample

In total, 741 cultures of *M*. *tuberculosis* complex were collected between 1999 and 2012 from patients with or without prior treatment history from 31 departments in Colombia; these isolates were obtained through systematic surveillance conducted by the INS. The isolates were stored in the mycobacteria group biobank. For analysis, the sample was divided into two equal 7-year periods for determining the change in genotypes. In total, 410 isolates were included in the first period, and 331 isolates were included in the second period.

### Ethics statement

All study procedures were approved by the Ethics Committee in Research (ECR). This study did not require informed consent. The isolates were obtained from the biobank of the mycobacteria group and used directly because of the surveillance function of the INS, which is the highest public health authority in Colombia.

### Methods for the microbiological study

#### Culture and identification

Isolates grown on Ogawa-Kudoh medium were sent to the Departmental Secretaries of Health of Colombia and analyzed for the species identification following the methodology described in the procedural handbook of the National Reference Laboratory of the INS [12] and the Centers for Disease Control and Prevention (CDC) guidelines [13].

#### Susceptibility testing for first-line drugs

The susceptibility testing for first-line drugs was performed using the simplified methodology of multiple proportions of Canetti Rist and Grosset [14]; the automated Bactec MGIT 960 Beckton Dickinson USA methodology was used.

### Study methods for molecular epidemiology

#### DNA extraction

The isolates identified as *M*. *tuberculosis* complex were reseeded in Lowenstein Jensen medium and incubated for 15 days at 37°C. DNA extraction was then performed as described by Van Soolingen et al. [15].

#### Genotyping (spoligotyping) of the DR locus

The DR locus was genotyped (spoligotyped) following the standard methodology described by Kamerbeer et al. [16].

The genotypes obtained by spoligotyping were translated into a binary code, and the octal code was compared with the SPOLDB4 international database of the Pasteur Institute of la Guadalupe (http://www.pasteur-guadeloupe.fr:8081/SITVITDemo online version) to determine the SIT (spoligo international type), family and international location [4].

The genotypes obtained were subjected to a grouping analysis using Bionumerics version 6.0 software (Applied Maths). A grouping was defined as three or more isolates having an identical pattern.

### Statistical analysis

A descriptive analysis of each variable was performed during each of the periods. The measures of central tendency and their 95% confidence intervals were calculated and compared to observe their tendencies. A bivariate analysis was performed, and the associations among the variables, the phenotypes of drug susceptibility, and the genotypes of the DR loci or families were determined using Epi Info 7.0 (CDC, public domain). The prevalence ratios and their 95% confidence intervals were estimated using Pearson’s chi-squared test or Fisher’s exact test. p<0.05 was considered statistically significant.

## Results

### Demographic and epidemiological descriptions

#### Sex and age

In total, 39.14% (n = 290) of the patients were female with an age range of 6 to 92 years, whereas 60.86% (n = 451) of the patients were male with an age range of 4 to 95 years. In the overall study population, 1.52% (n = 11) of the patients were between the ages of 1 and 15 years, 36.33% (n = 263) were between the ages of 16 and 30 years, 31.49% (n = 228) were between the ages of 31 and 45 years, 21.13% (n = 153) were between the ages of 46 and 60 years, and 9.53% (n = 69) were older than 60 years. However, no data were available on 2.29% (n = 17) of the patients.

#### Origin

The isolates included in the study were from patients from all of the departments in Colombia except San Andrés and Vaupés. The Department of Valle del Cauca contributed 33.6% of the isolates in this study S1 Fig.

#### State of TB treatment

In total, 22.4% (n = 166) of the isolates were from previously treated patients, whereas the remaining 77.6% (n = 575) were from patients who did not have a previous history of treatment.

### Phenotype of isolates susceptible to first-line drugs

In total, 61.94% (n = 460) of the isolates were resistant to one or more drugs, and 33.33% (n = 246) of the isolates were susceptible. However, susceptibility information was not available for 4.72% (n = 35) of the isolates. The MDR phenotype was found in 19.83% (n = 147) of the isolates. The patterns of resistance to first-line drugs are described in Table 1.

**Table 1:** Distribution of patterns of susceptibility to first-line drugs.

Resistance Pattern	N	%
H+R+S+E	66	8.91
H+R+S	59	7.96
H+R+E	5	0.67
H+R	17	2.29
H+S+E	12	1.62
H+S	90	12.15
H+E	4	0.54
R+E	1	0.13
S+E	1	0.13
S+R	1	0.13
E	8	1.08
H	78	10.53
R	3	0.4
S	115	15.52
Sensitive	246	33.2
No data	35	4.72
TOTAL	741	100

H: Isoniazid, R: Rifampicin; S: Streptomycin: E: Ethambutol.

### Genotypes by spoligotyping and drug susceptibility

In total, 170 genotypes were identified; the SIT42 (LAM 9) was the most frequent genotype and included 24.7% (n = 183) of the isolates. The distribution of the families identified, their frequency and the susceptible phenotypes are presented in Table 2.

**Table 2:** Distribution of the identified genotypes by family and frequency among the drug-susceptible phenotypes.

FAMILY	SIT	Total		Resistant		Sensitive		No data
		N	%	N	%	N	%	N
Beijing	190	23	3.1	23	100	0	0	
Beijing	1	1	0.13	1	100	0	0	
CAS1_DELHI	26	1	0.13	0	0	1	100	
H	62	68	9.18	34	50	34	50	
H	50	33	4.45	18	54.55	14	42.42	1
H	OTHERS	28	3.78	19	67.86	9	32.14	
H	727	14	1.89	5	35.71	9	64.29	
Orphan	NR	126	17	91	72.22	32	25.4	3
LAM	42	183	24.7	116	63.39	63	34.43	4
LAM	OTHERS	45	6.07	27	60	15	33.33	3
LAM	33	18	2.43	11	61.11	7	38.89	
LAM	20	15	2.02	11	73.33	4	26.67	
LAM	17	19	2.56	16	84.21	3	15.79	
LAM	130	11	1.48	9	81.82	2	18.18	
LAM	162	9	1.21	7	77.78	2	22.22	
M bovis	820	1	0.13	0	0	1	100	
MANU S	54	4	0.54	0	0	0	0	4
S	34	7	0.94	4	57.14	3	42.86	
S	831	1	0.13	1	100	0	0	
T	53	51	6.88	17	33.33	23	45.1	11
T	OTHERS	23	3.1	10	43.48	13	56.52	
U	881	20	2.7	14	70	6	30	
U	OTHERS	10	1.35	9	90	1	10	
U	106	8	1.08	7	87.5	1	12.5	
U	523	9	1.21	0	0	0	0	9
X	91	5	0.67	2	40	3	60	
X	OTHERS	8	1.08	7	87.5	1	12.5	
TOTAL		741	100	460	62.08	246	33.2	35

### Description of groupings by spoligotyping

**Fig 1:**
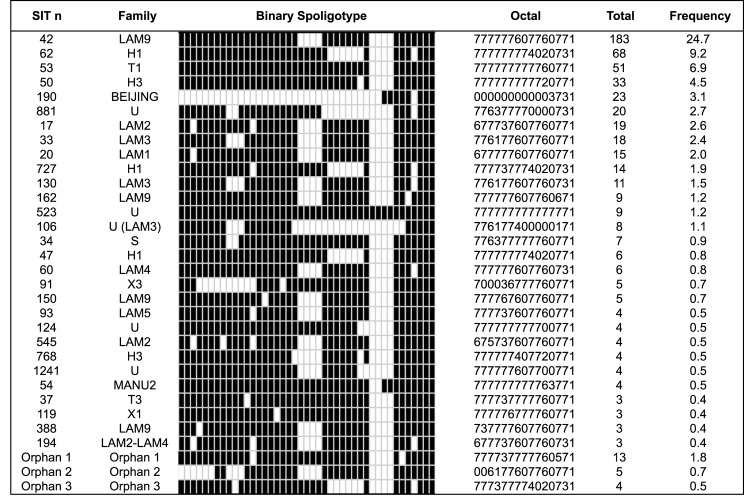
Major genotypes identified according to family and frequency in Colombia, 1999–2012.

There were 32 groupings that consisted of 3–183 isolates from the total population; therefore, 79.8% (n = 591) of the isolates were grouped. The major grouping genotypes were SIT42 (LAM9), SIT62 (H1), SIT53 (T1) and SIT50 (H3) (Fig 1).

For both study periods analyzed (1999–2005 and 2006–2012), a high proportion of grouped isolates was found, (78.78% (n = 323) and 80.97% (n = 268), respectively).

In total, 80.4% (n = 369) of the resistant isolates were grouped together, whereas 77.7% (n = 192) of the susceptible isolates were grouped together. The two populations showed no significant differences in terms of active or recent transmission (p = 0.229).

### Bivariate analyses and associations

The results from analyzing the variables according to the presence of grouping isolates are shown in Table 3. The variable genotypic family, specifically the Haarlem family, was associated with grouping isolates (p = 0.031), whereas the T, X and Orphan families were associated with the non-grouped isolates (p < 0.001).

**Table 3:** Bivariate analysis of the variables associated with grouping isolates.

Variables		Grouping				p*
		Yes		No		
		n	%	n	%	
Sex						
	Male	357	79.3	93	20.6	
	Female	234	80.4	57	19.6	0.397
Age						
	0–15 years	10	90.9	1	9.1	0.21
	16–30 years	215	83	44	71	0.137
	31–45 years	167	76.6	51	23.4	0.385
	46–60 years	118	80.3	29	19.7	0.243
	61–75 years	28	65.1	15	34.9	0.379
	>76 years	18	72	7	28	ref
Status of treatment						
	PT	132	81.9	29	18.1	
	NT	459	79.1	121	20.8	0.24
Susceptibility to first-line drugs						
	R	369	80.4	90	19.6	
	S	192	77.7	55	22.3	
	ND	30	85.7	5	14.3	0.229
MDR						
	Yes	125	85	22	14.9	
	No	466	78.4	128	21.5	0.045
Period of the study						
	1999–2005	323	78.8	87	21.2	
	2006–2012	268	77.7	63	19	0.26
Family						
	LAM	280	93.3	20	6.7	ref
	Beijing	23	95.8	1	4.8	0.527
	U	45	95.7	2	4.3	0.51
	S	7	87.5	1	12.5	0.435
	H	125	87.4	18	12.6	0.031
	X	8	61.5	5	38.5	0.001
	T	54	72.9	20	27.1	<0.001
	Orphan	45	35.7	81	64.3	<0.001
	MANU	4	100	0	0	NA
	M. bovis	0	0	1	100	NA
	CAS1_Delhi	0	0	1	100	NA

* Test of significance: Fisher’s Exact

Moreover, a statistically significant association was found between the MDR isolates and the non-grouped isolates (p = 0.045).

The variables analyzed regarding the resistance to first-line drugs are shown in Table 4. The Beijing family was strongly associated with drug-resistant isolates, whereas the Haarlem (p = 0.003) and T (p < 0.001) families were associated with susceptibility to first-line drugs.

**Table 4:** Bivariate analysis of the variables associated with drug resistance.

Variables		Drug resistance				p*
		Yes		No		
		n	%	n	%	
Sex						
	Male	276	65.7	144	34.3	
	Female	182	63.9	103	36.1	0.334
Age						
	0–15 years	8	72.7	3	27.3	0.285
	16–30 years	156	61.7	97	38.3	0.363
	31–45 years	141	66.2	72	33.8	0.212
	46–60 years	95	66	49	34	0.229
	61–75 years	32	74.4	11	25.6	0.098
	>76 years	14	56	11	44	ref
Status of treatment						
	PT	146	90.7	15	9.3	
	NT	313	57.4	232	42.6	<0.001
Grouping						
	Yes	369	65.8	192	34.2	
	No	90	62.1	55	37.9	0.229
Period of the study						
	1999–2005	326	79.9	82	20.1	
	2006–2012	133	44.6	165	55.4	<0.001
Family						
	LAM	197	67.2	96	32.8	ref
	Beijing	9	69.2	4	30.8	0.527
	U	5	62.5	3	37.5	0.522
	S	91	73.9	32	26.1	0.105
	H	30	78.9	8	21.1	0.098
	X	76	53.5	66	46.5	0.003
	T	27	42.9	36	57.1	<0.001
	Orphan	24	100	0	0	
	MANU	0	100	1	0	NA
	M. bovis	0	0	1	100	NA
	CAS1_Delhi	0	0	1	100	NA

* Test of significance: Fisher’s Exact

Moreover, the isolates from the second study period were associated with drug sensitivity (p < 0.001), and the resistant isolates were associated with patients who had been previously treated (p < 0.001).

### Trend analysis

#### Families

**Fig 2:**
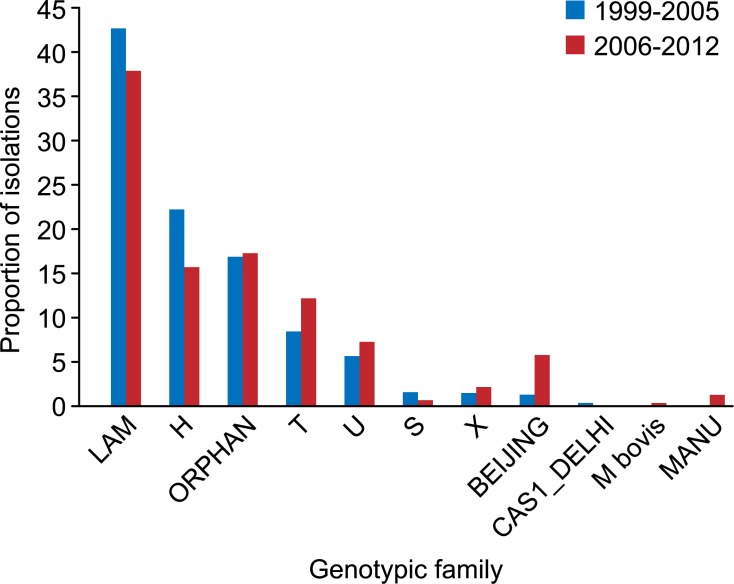
Distribution of the genotypic families according to the isolation period.

The evaluation of the dynamics of presentation of the genotypic families in the total study population showed a significant increase in the Beijing family from the first to the second study period (p < 0.001). Similarly, the T family showed a significant increase during the second period (p = 0.03) (Fig 2).

**Fig 3:**
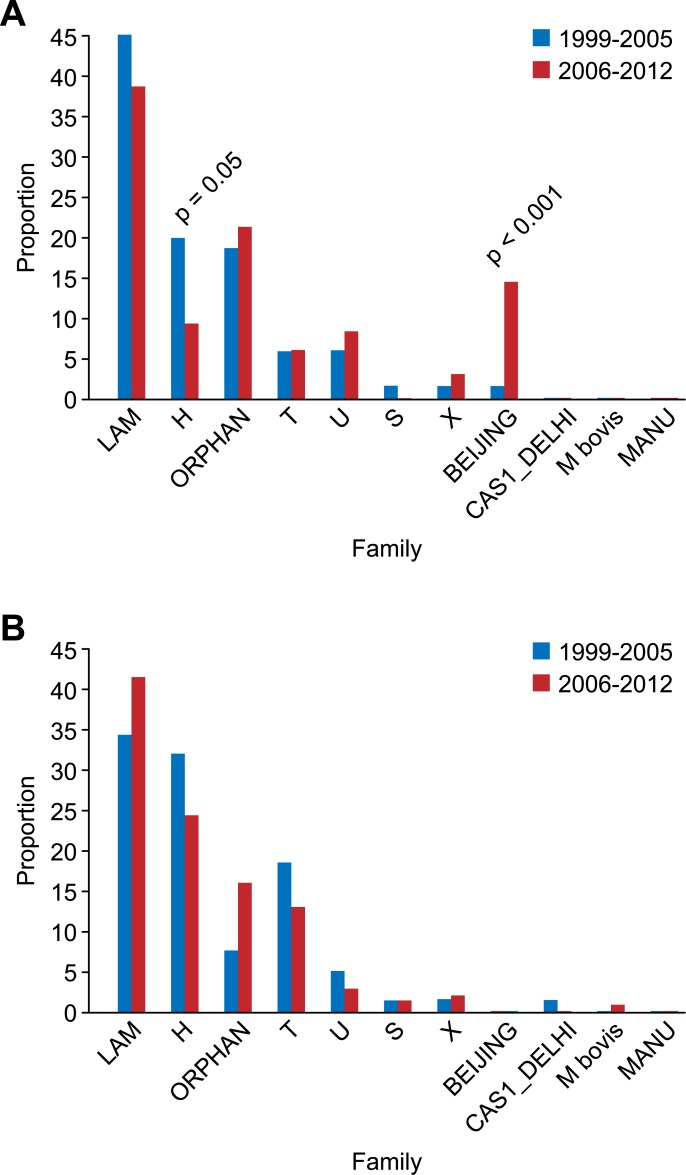
Transmission dynamics of genotypes in isolates resistant (A) and sensitive (B) to first-line drugs.

The proportion of drug-resistant isolates of the Beijing family increased significantly in the second study period compared with the first study period (p < 0.001); conversely, the Haarlem family proportion was significantly reduced (p = 0.05) during the same period. This behavior was most likely determined by the MDR isolates (Fig 3A). Significant variation among the susceptible isolates was not observed in any of the circulating families in Colombia when comparing the two periods (Fig 3B).

#### Grouped isolates

No significant difference was found in the total population when the proportion of grouped isolates from the first period was compared with that from the second period (p = 0.260).

No significant differences were found (p = 0.44) in the drug-resistant population, when comparing the proportion of grouped isolates from the first study period (80.1%, n = 261) with that from the second period (81.2%, n = 108).

Moreover, when comparing the proportion of grouped isolates from the first study period (65%, n = 61) with those from the second period (66%, n = 36), no significant differences were found (p = 0.5) in the MDR population. Similarly, when comparing the proportion of grouped isolates from the first study period (73.1%, n = 60) with those from the second period (80.00%, n = 132), no significant differences (p = 0.146) were found in the susceptible population.

## Discussion

Since the introduction of spoligotyping in 1997, this method has become one of the most widely used tools worldwide for typing of isolates of *Mycobacterium tuberculosis* because it adds discriminatory power to previously existing tools, such as RFLP IS6110 (reference method), used for molecular epidemiology studies of tuberculosis [17–19].

Given the evolutionary mechanisms of the DR region (i.e., the sequential loss of spacers without the ability to recover lost spaces), the unambiguous phylogenetic classification of strains according to patterns or spoligotypes enables the strains to be related to specific phenotypes of individual clinical isolates. This method has increased the understanding of the population genetics of *Mycobacterium tuberculosis*, its evolutionary history and transmission in different regions. Identical genotypes are considered to be isolates that cause active transmission, whose quantification enables the measurement of the effect of strategies for tuberculosis control programs with the aim of reducing and controlling disease transmission.

In this framework, the present study demonstrated that in Colombia between 1999 and 2012 approximately 80% of the isolates belonged to groups suggesting that the program strategies to control TB probably was slightly affected; this situation should be corroborated using the combination of highly discriminating methods, this high grouping is a much higher proportion than that reported in other countries where there are effective control programs. A national study in the United States reported that 34.4% of isolates were grouped during the period of 2008 to 2010 [20]. Similar proportions of grouped isolates were reported in this study and in countries with a high burden of disease, including some of the African countries belonging to the group of 22 countries selected by the WHO to emphasize control strategies for their high levels of TB [21]. Consistent with our data, previous studies in Colombia have reported a high proportion of *M*. *tuberculosis* groupings using genetic methods for different regions; in particular, grouping proportions between 20 and 74% have been reported [22–24]. Each region must expand the number of isolates characterized to determine the own genotypes. One strength of this work is the characterization of a large number of isolates (741) from 31 of the 33 departments in Colombia over a long study period.

A comparison of the two seven-year periods emphasizes that the status of TB transmission has not changed; this observation agrees with reports of classical epidemiology in which no variation was observed in the number of new cases diagnosed over time in Colombia [1].

There were no significant differences in the MDR or sensitive isolates in the two periods studied with respect to the groupings, which may indicate that the level of active transmission is not decreasing in Colombia.

The Haarlem family was associated with grouped isolates, whereas the T, X and Orphan families were associated with the non-grouped isolates (p <0.001), which most likely represented endogenous reactivation or latent tuberculosis.

In this study, a high genetic diversity of *M*. *tuberculosis* was reported, with 170 different genotypes present that were mainly represented by four families: LAM (39.9%), Haarlem (19%), Orphan (17%) and T (9%). The isolates of type SIT42 were the most common isolates belonging to the LAM9 family, which was found in all the departments included in this study. The SIT62 (H1) was the second most common type of isolate Fig 1. The LAM family has been described as prevalent in other countries including Paraguay and Venezuela and in countries in the Americas, Europe and the Caribbean [25, 26]. Previous studies that genotyped isolates in Colombia using spoligotyping reported that the LAM family was the most frequent family in regional circulation, followed by the Haarlem family [24–27]. Moreover, in this study the MANU family is first report in Colombian isolates from individuals coinfected with HIV.

In total, 83 genotypes were found that had not been previously reported (orphans). These genotypes were likely native from Colombia, and 72.2% of the newly discovered genotypes were resistant to one or more drugs Fig 3A and 3B. The high proportion of patterns and orphan spoligotypes detected in this study, particularly those belonging to new cases, indicates that these genotypes should be monitored and investigated further because they may have been generated by recent developments in pre-existing genotypes.

Regarding the association of families with phenotypes susceptible to first-line drugs, it was shown that an isolate of the Beijing family was a predictor of drug-resistant insulation; the frequency of these isolates increased significantly during the second period of the study. Previous studies showed that some isolates of the Beijing family are sensitive to drugs [28] and that in the Latin American population, Beijing family isolates are rare [29]. It is important to emphasize that all of our isolates belonging to the Beijing family were resistant to first-line drugs and were exclusively obtained from the municipality of Buenaventura, Valle del Cauca, which has an African-American population [30]. It is known that certain ethnic characteristics confer susceptibility to human hosts for infection and disease development by strains of this family, which was most likely determined by the co-evolution of the pathogen and the population group [31].

The genotypes SIT53 (H) (p = 0.003) and SIT727 (T) (p <0.001) were clearly associated with isolates sensitive to first-line drugs. This finding has also been documented in recent studies from Taiwan [32,33]; therefore, it would be useful to continue monitoring the presentation of these genotypes over time to predict the success of the treatment schemes used in Colombia. It is necessary to intensify to the epidemiological surveillance of drug-resistant tuberculosis in Colombia, because we find 31.9% of isolates that were MDR, 44.6% of the isolates were mono-resistant and 20.9% of the isolates were bi-resistant during this 14-year period. Because the treatment schemes used in Colombia are conjugated, it is assumed that the isolates with mono- and bi-resistance to first-line drugs would have been eliminated by these schemes; therefore, it is believed that these isolates reflect unfinished treatments and dropouts resulting from lack of adherence to treatment by Colombian patients.

In summary, based on the results of this study, molecular markers such as MIRU-VNTR [34] should be used to increase the power of discrimination and to identify the real proportions of groupings associated with active transmission in Colombia while recognizing the benefits of the knowledge of the genotypes circulating in Colombia by spoligotyping. This information can help the National Tuberculosis Control Program intensify its intervention strategies to achieve early detection and timely establishment of treatment for cases of active tuberculosis because the delay in treatment is a key factor of disease transmission. This action is proposed because the drug-resistant isolates have not been shown to be responsible for the active transmission of TB in Colombia.

This study provided an overview of the population structure of *M*. *tuberculosis* in all regions of Colombia and may be the first national study of genetic diversity identified by spoligotyping and its association with susceptibility and the active/recent transmission of tuberculosis in Colombia.

As Colombia strives to eliminate tuberculosis, surveillance of genotypes may lead to earlier detection of micro-epidemics and outbreaks, resulting in continuous improvement of TB control activities and maximizing the use of the limited resources of the state public health system both locally and nationally.

## Supporting Information

S1 Fig Origin of the isolates included in the study. (EPS)
